# Skincare in Rosacea from the Cosmetologist’s Perspective: A Narrative Review

**DOI:** 10.3390/jcm12010115

**Published:** 2022-12-23

**Authors:** Danuta Nowicka, Karolina Chilicka, Iwona Dzieńdziora-Urbińska, Renata Szyguła

**Affiliations:** 1 Department of Dermatology, Venereology and Allergology, Wrocław Medical University, 50-368 Wrocław, Poland; 2 Faculty of Health Sciences, University of Opole, 45-060 Opole, Poland

**Keywords:** rosacea, cosmetics, cosmetology, professional treatment, patient education

## Abstract

Rosacea is a common skin disease that affects about 5% of the general population. Its symptoms include telangiectasia, persistent erythema, burning/stinging sensation, dry skin sensation, and pruritus. It is characterized by a chronic course with frequent exacerbation. It often coexists with anxiety and depression, reducing the quality of life of affected patients. The etiopathogenesis of rosacea is complex and not fully elucidated; hence, there is no causative effective treatment. In this review, we highlight the role of a cosmetologist in the treatment of rosacea and the maintenance of remission. As part of medical treatment, patients are advised to introduce lifestyle changes and use proper skin care; a cosmetologist can help educate patients affected with rosacea, create effective home care programs for skin care, and support them with treatments in beauty salons. Proper skin care is essential, including the use of dermocosmetics, cleansing of the skin, and frequent visits to beauty salons for tailored apparatus procedures. A cosmetologist is more accessible to patients and can help implement healthy daily habits, including skin care and eating habits, as well as support and mediate good communication between the patient and the patient’s treating physician, thereby improving compliance and ensuring long-term satisfactory outcomes.

## 1. Introduction

Rosacea is a common skin disease with characteristic symptoms such as telangiectasia, persistent erythema, burning/stinging sensation, dry skin sensation, and pruritis [1]. It can be accompanied by the presence of small pus-filled bumps. Asymptomatic periods are interrupted by frequent exacerbations that can last from a couple of weeks to months. Rosacea can be mistaken for acne, other skin problems, or natural ruddiness [2]. Rosacea can affect anyone. A meta-analysis showed that the prevalence of rosacea is 5.46% in the general population and 2.39% in dermatological outpatients [3]. This skin condition affects women slightly more frequently than men and decreases with age. The prevalence reaches 13.5% in people aged 18–25 years, 9.6% in people aged 26–54 years, and only 1.0% in people aged 55 years and older. The occurrence of unpleasant and uncomfortable skin sensations is more frequent in women than in men (26% vs. 18.3%) [4]. Data on incidence are limited. A systematic review on incidence of rosacea identified only one study from the UK reporting the incidence of 1.65 per 1000 person-years [3]. Previously, rosacea was classified according to subtypes; however, the current guidelines recommend a phenotype approach that is believed to more accurately address patient characteristics and help better choose personalized treatment [5,6]. Until now, no causative treatment for rosacea has been developed; however, currently available treatments can help control the disease and reduce its signs and symptoms [2]. This condition is not life-threatening; however, due to the chronic course, the signs visible on patient’s face, and the lack of effective treatment, it is often accompanied by anxiety and depression, which is a burden for affected patients [1,7,8].

The care of rosacea skin is a challenge for a cosmetologist. This chronic inflammatory disease has multifactorial etiopathogenesis, including the involvement of the immune and neurovascular systems. In addition, an interplay of risk factors and clinical symptoms can reduce patient quality of life, which, in turn, can worsen the course of the disease and affect patient functioning [2]. For the treatment of rosacea, it is important to build a good relationship and trust between the treating physician/cosmetologist and the patients. This is because symptoms can be triggered by some environmental factors or lifestyle habits. Patients can modify their lifestyles and avoid harmful factors if they have a good knowledge of risk factors and are motivated to do so.

## 2. Methods

We conducted a review of the literature to provide an up-to-date overview of the research on rosacea and the treatment of rosacea from the cosmetologist’s perspective. Such a perspective is often overlooked in the scientific literature; however, proper skin care and lifestyle are part of a successful approach to rosacea. The results are presented in the form of a topical review in which the selection of the content is based on the authors’ experience.

## 3. Food Items and Other Triggering Factors for Flares of Rosacea

In 2022, the National Rosacea Society conducted a survey and collected responses from 1066 patients with rosacea [9]. The aim of this survey was to investigate which factors can trigger or aggravate the symptoms of the disease. The most important factors were sun exposure, emotional stress, and hot water. The survey pointed out the importance of diet and a wide variety of substances that can cause flares such as alcohol, spicy foods, certain fruits and vegetables, marinated meats, and dairy products. The evidence on food items that are responsible for disease flares comes from small studies or case reports [10]. Food triggers include figs, bananas, plums, chocolate, cheese, yoghurts, cream, soy sauce, eggplant, spinach, beans, peas, and broad beans. Regarding drinks, strong and hot coffee or tea, alcohol, and sweeteners added to drinks can act by activating transient receptor potential ion channels leading to functional impairment of the skin barrier. Although some patients with rosacea indicate that coffee has the potential to worsen symptoms, a large study provided opposite conclusions. The study by Li et al. found that 82,737 women with rosacea who consumed more caffeine had a lower risk of incident rosacea, highlighting its possible protective effect [11]. Nevertheless, patients should be informed that the temperature of the beverage and byproducts are equally important for flares as the main ingredients. Furthermore, the consumption of histamine-rich foods and sugary foods can also lead to rosacea exacerbations [12].

Food items and diet can also have a positive impact on the rosacea course. Daily supplementation of omega-3 acids contributes to a reduction in inflammation and the prevention of telangiectasia [13,14]. In addition, the diet should be easily digestible, alkaline, and rich in products containing fiber, as well as vitamins C, PP, and B2 [15]. Lastly, patients with rosacea often suffer from gastrointestinal diseases. Supporting a healthy gut microbiome can translate into improving general functioning in this group of patients [12].

## 4. Role of Microorganisms in the Development of Rosacea

The importance of microbes in the pathogenesis of rosacea is still controversial. A constant interaction among the skin, microorganisms, and the environment leads to a disturbed balance of the microflora, which in turn leads to the development of dermatoses. Pathogens such as *Demodex folliculorum, Bacillus oleronius, Helicobacter pylori, Staphylococcus epidermidis*, and *Chlamydophila pneumoniae* play the greatest role in the development of rosacea [16,17]. Many of the abovementioned microorganisms belong to the human physiological flora; therefore, it is difficult to define their role in the development of the disease. Microorganisms residing on the surface of human skin interact closely with the immune system and protect the host against the attack of infectious agents.

The composition of the skin microbiome depends on various factors such as sex, age, and comorbidities, as well as the environmental conditions of the skin, pH, humidity, or lipid composition [18,19]. Human skin microorganisms show the presence of receptor ligands for keratinocytes. Toll-like receptor 2 (TLR2) and NALP3 receptor trigger the inflammatory cascade [20,21]. Stimulation of keratinocytes leads to the release of inflammatory factors such as cytokines and chemokines, as well as the stimulation of angiogenesis through the production of vascular growth factors and cathelicidin stimulated by serine protease [22,23]. Changes in the environmental conditions on the skin and the inflammation present there cause quantitative and qualitative changes in the human microbiome. These conditions favor the multiplication of *Demodex* spp. and cutaneous staphylococci *(S. epidermidis).* However, it should be mentioned that new microorganisms inhabiting the skin still stimulate the immune system, leading to dilation of blood vessels, intensification of chemotaxis, and, as a result, a change in the erythematous form of rosacea to a maculopapular form [17].

*D. folicullorum*, which belongs to the mite species, is believed to play an important role in the pathogenesis of rosacea. This mite is mainly associated with maculopapular and ocular forms [24]. It is considered to be a commensal microorganism; however, in some cases, it can activate inflammation and, thus, alter functions of the immune system. Chitin released from *D. folicullorum* has been shown to stimulate TLR2 in keratinocytes, which in turn leads to the development of inflammatory and erythematous changes and disorders of the sebaceous glands [25]. Research has shown that anti-*Demodex* antibodies occur mainly in people affected by rosacea. In addition, *D. folicullorum* is considered a cofactor of the inflammatory reaction in the body because the number of mites correlates with the level of activation of the immune system [21,25]. Moreover, the presence of *D. folicullorum* is found in the secretions from the sebaceous unit in a large percentage of patients with rosacea, while, in healthy subjects, this percentage is much lower. Overall, a higher concentration of the pathogen in hair-sebaceous units correlates with the occurrence of rosacea, such a relationship is not observed in healthy people [21,26]. Human *Demodex* spp. also has its own microbes, which consist of several to several dozen species of bacteria. The greatest amount of DNA of various bacterial strains was isolated from *D. folicullorum* bottling in patients with the maculopapular form of rosacea; it was slightly lower in patients with the erythematous form, while it was almost absent in healthy people. The most numerous bacteria living in the microbiota of *Demodex* mites are *Firmicutes, Proteobacteriae*, and *Actinobacteriae*, as well as *Bartonella quintana*. The last one was isolated from a patient with the erythematous form and is the etiological factor of root fever and endocarditis. *B. quintana* is transmitted by human lice, and it is believed that various species of mites, including *D. folicullorum*, may be a vector for the transmission of infection, but there is no evidence that *B. quintana* influences the development of rosacea [27,28]. However, one of the *Demodex*-inhabiting species, *Bacillus oleronius*, is believed to play a role in the pathogenesis of rosacea. *B. oleronius* antigens have been shown to initiate inflammation, and two peptides, 83 and 62 kDa, are highly immunogenic [29]. Bacterial proteins have the ability to activate neutrophils, release metalloproteinases, and synthesize cathelicidin. Proinflammatory cytokines are released, e.g., tumor necrosis factor (TNF)-∝ and interleukin (IL)-8, stimulating the development of inflammation around the hair follicle [30]. Additionally, bacterial liposaccharides cause the destruction of the hair follicle wall, negatively affecting the migration of epidermal cells, and, in the case of eyelash involvement in the eye form, they damage the corneal epithelium [16,29]. A positive correlation was also demonstrated between serum reactivity to *B. oleronius* antigens and the erythematosus-vascular form of rosacea [31]. A number of scientific studies also indicate the role of *S. epidermidis* in rosacea [32]. In the maculopapular form of rosacea, bacteria have been observed to grow and secrete proteins that stimulate the immune system, which has not been observed in the *S. epidermidis* strains that are found in healthy people. This is a consequence of the exacerbation of inflammation in rosacea [33].

Pathogenic bacteria that infect internal organs can also contribute to the development or exacerbation of skin diseases. There are reports on the role of *H. pylori* in the pathogenesis of skin diseases, including rosacea [34,35]. Stimulation of gastrin secretion by *H. pylori* contributes to paroxysmal erythema, which is part of the clinical picture of rosacea. After *H. pylori* eradication, it was found that the skin condition of patients with rosacea improved significantly; therefore, it is assumed that the Cag A cytotoxin characteristic of this bacterium may be a factor that exacerbates the course of rosacea [36]. Research shows that the Cag A gene was present in 67% of patients with rosacea and that reactive antibodies to the CagA cytotoxin were detected in 75% of patients with rosacea. However, at present, the role of *H. pylori* infection in rosacea is rather negated [34]. Chronic infection by *Chlamydia pneumoniae* may also play a role in the pathogenesis of rosacea. *C. pneumoniae* antigens were found in 40% of skin biopsies in patients with rosacea, and reactive antibodies against *C. pneumoniae* were present in the blood in 90% of cases [37].

## 5. Microbiome and Its Role in Rosacea

The term microbiome denotes the collection of genomes of all microorganisms that inhabit the human body. Microbiota is a collection of microorganisms as cells, while microflora is an old term denoting the total of living microorganisms in a given environment. Currently, it is used for the population of bacteria itself (bacterial microflora) [38]. For many years, research has been conducted on the human microbiome, demonstrating that it plays an important role in maintaining systemic homeostasis through the ability to metabolize food substances, increase the absorption of minerals, produce B and K vitamins, prevent intestinal colonization by pathogenic bacteria, reduce inflammatory processes, inactivate toxins and carcinogens, and stimulate of the maturation of cells of the immune system. Intestinal dysbiosis leads to the development of many lifestyle diseases, including acne and rosacea [38]. The compositions of skin microbiota can change with age and during the course of the disease. An imbalance in skin microbiota can also contribute to skin pathologies [39]. The human microbiome consists of approximately 30 trillion microorganisms and 3.3 million microbial genes, which are responsible for the proper functioning of the human immune system [40]. The intestinal microbiome comprises about 90% of bacteria belonging to *Bacteroidetes* and *Firmicutes*, with the remainder comprising *Actinobacteria*, *Proteobacteria*, and *Verrucomicrobia* [38]. The results of the conducted research emphasize the diversity of the microbiome in patients with rosacea (Table 1).

## 6. Skin Care in Rosacea

The use of dermocosmetics is of great importance in the daily care of skin with rosacea. These are preparations intended for a specific type of skin, and their main purpose is to prevent and reduce skin ailments. They combine the properties of a drug and a cosmetic. Patients with rosacea often experience problems with tolerance to cosmetics; therefore, it is important that the selected cosmetic is intended for rosacea skin. Otherwise, there is a high risk of exacerbation of the disease [48]. The case–control survey on skin care habits compared the skin care patterns of 1245 people with rosacea with 1538 people without skin problems [49]. The survey found factors that contributed to the development of rosacea. These were using foaming cleansers, makeup more than six times a week, facial masks more than four times per week, facial treatments in beauty salons more than once per week, and beauty salon products. Moisturizing products and sunscreen creams had protective effects. 

The composition of dermocosmetics is of great importance, as selected active substances and vehicle types can either heal skin lesions or contribute to the exacerbation of skin problems [50]. Vitamin C plays a key role because it has a protective and antioxidant effect, and it neutralizes free radicals. Additional advantages include brightening and antiaging properties, as well as strengthening blood vessels and reducing redness [51,52]. Vitamin K has a sealing effect on blood vessels and reduces erythema. The main action of vitamin PP is the inhibition of histamine secretion, which is responsible for the dilation of blood vessels and the intensification of erythema. It also reduces inflammatory processes and has an anti-swelling effect. Its advantage is that it can be used in light emulsions because it dissolves well in water. Allantoin and D-panthenol are very often used in dermocosmetics because they have a soothing and healing effect. Bioflavonoids are responsible for the regeneration of blood vessels, and they have astringent, anti-inflammatory, and anti-swelling properties. The use of flavonoid licochalcone has been shown to reduce erythema in patients with pre-rosacea and rosacea [53]. Essential unsaturated fatty acids are responsible for regulating the permeability of the stratum corneum, thus reducing inflammatory processes. Using them in daily care increases the elasticity of the skin, thus enhancing its protective functions against various negative factors [15]. Ceramides play a very important role in keeping the skin at a constant temperature, which is a very desirable effect. One should not forget about acids, which are used in various skin problems; however, in the case of rosacea, we use polyhydroxy acids (gluconolactone) and bionic acids (lactobionic, maltobionic, and cellobionic). Acids not only improve the functioning of the epidermal barrier, but also protect the skin against ultraviolet rays and free radicals, as well as smooth the surface of the skin. Retinaldehyde is a substance that improves the thickness of the epidermis, reduces erythema and the amount of telangiectasia, and reduces inflammation. In color cosmetics, silicon dioxide is very often used, which, thanks to its green color, is used as a preparation that camouflages telangiectasia. Tissue metalloproteinase inhibitors (TIMPS) are present in algae extracts. Their main action is to prevent skin thinning, prevent blood vessels expansion, and positively influence the inhibition of inflammatory processes. 

A variety of factors should be avoided in skincare. The patient with rosacea should avoid using soaps, cosmetic preparations with alcohol, skin-drying preparations, and fine and coarse peelings. Sunscreens are recommended to protect the skin against UV radiation which is one of the most important factors responsible for triggering factors for flares of rosacea; however, chemical filters are not recommended because they can irritate the surface of the epidermis and aggravate the disease. Instead, mineral filters are recommended that do not irritate the skin and contain titanium and zinc oxide and dioxide. Furthermore, physical filters have the advantage of being colored to mask erythema and other skin eruptions [51]. Cosmetics used should aim to protect the skin against triggering factors such as ultraviolet radiation from the sun, wind, and pollutants, as well as cold and hot temperatures [2].

For skin care at home, not only is the composition of cosmetics important, but so is the frequency of using them. The skin should be cleansed every day to remove triggering molecules from the environment. However, for everyday face care and cleansing, dermocosmetics for sensitive skin are recommended, containing moisturizing and softening substances that do not destroy the protective lipid layer [54].

## 7. Cosmetology Treatments

Patients with rosacea are common clients of many beauty and cosmetology salons. For this reason, procedures should be in place on how to proceed with patients affected by this skin condition. In the first step, the patient must go through a cosmetic interview, determining the indications and contraindications for treatments and the frequency of treatments [49]. Furthermore, various treatment techniques can be combined to diversify and enhance the effects; however, some combinations are not recommended in the treatment of rosacea [48].

Several types of light and laser treatment have been proven to be effective in the treatment of rosacea symptoms; however, their effectiveness and safety profile differ depending on the characteristics of the patient. Intense pulsed light (IPL) therapy with a wavelength of 560 nm is indicated to reduce telangiectasia and erythema, as well as papules and pustules. The treatment alleviates inflammation, itching, swelling, burning, and pain. Hemoglobin is the chromophore, and the action of IPL is based on photothermolysis or thermal damage to the vessels, which provides the effect of intravascular coagulation [2,55]. The use of a single IPL treatment in combination with a topical skin care regimen can produce a significant, long-term reduction in overall facial redness. More than 80% of patients undergoing IPL claim to be satisfied or very satisfied with treatment. The procedure is safe; however, transient burning may occur after the procedure [56]. Although good results can be achieved after a single treatment, experts recommend multiple sessions at intervals of 1–3 weeks [2]. Other types of light therapy are also effective. Pulsed dye laser (PDL) with a wavelength of 595 nm is indicated in the occurrence of telangiectasia and erythema. The use of longer pulses reduces discoloration, as well as produces longer periods without exacerbations [57]. The use of a neodymium:yttrium–aluminum–garnet laser (Nd-YAG) with a wavelength of 1064 nm shows effectiveness in the treatment of papules and pustules and in reducing telangiectasia. It is the best choice of other light-based therapies for patients with rosacea with large and deep telangiectasias and is considered to be a good option for patients with dark skin. Longer pulses of this laser deliver equivalent energy at a slower rate uniformly and gently in comparison to short pulse durations; thus, Nd-YAG is also a good option for patients suffering from easy bruising [58]. It was also found that using the “in motion” technique diminishes side-effects in patients with rosacea [59]. Potassium titanyl phosphate (KTP) with a wavelength of 532 nm is the most suitable for patients with superficial and thin telangiectasias. This laser is the best option for people with fragile capillaries who easily develop bruises. On the other hand, it is not recommended for skins with higher phototypes, as there is a risk of skin discoloration [58,60]. Pro-yellow laser with a wavelength of 577 nm laser is a novel treatment with little evidence. The application of this treatment resulted in a reduction in symptoms of erythematotelangiectatic rosacea. In addition, the treatment was very well tolerated [61]. Furthermore, a pro-yellow laser was shown to reduce the density of *D. folliculorum* and *D. brevis* on the skin of patients with rosacea [62]. A light-emitting diode (LED) modifies cellular activity, which results in the anti-inflammatory effect by producing from low-intensity nonthermal irradiation. This type of treatment is indicated in erythematous and inflammatory lesions [2]. Its effectiveness was explained in an experimental model that reported that LED downregulates cathelicidin, kallikrein, and TLR2 expressions in keratinocytes and rosacea-like mouse skin [63]. Although light and laser therapy is safe and can be used in many patients when chosen carefully, it has some contraindications such as active skin inflammation, cardiovascular failure, venous thrombosis, treatment with photosensitizing drugs, cancer, pregnancy, lactation, implanted pacemaker, autoimmune diseases, skin photoallergies, herpes simplex infection, a fresh tan (at least 8 weeks waiting period from the last sunbathing is recommended), and tuberculosis [61,64,65].

Low-density micro-focused ultrasound can reduce signs of erythematotelangiectatic rosacea [66]. After a single treatment, over 90% of the patients reported improvement that was maintained for up to 1 year. The procedure is generally safe; however, transient side-effects such as bruising, tenderness, and redness develop in one-third of treated patients. 

Some researchers attempted to introduce active substances through the skin in order to reduce the symptoms of rosacea. A variety of techniques can be used for this purpose such as intradermal microinjections (mesotherapy), microneedling, sonophoresis, and ultrasound. These treatments increase the penetration of active substances into the skin. Several substances have been investigated so far, including antifibrinolytic agents (tranexamic acid), antioxidants and angioprotectors (vitamin C), organic silica, amino acids, and hyaluronic acid [2,67].

Although many treatments bring good results, some other technologies should be avoided in patients with rosacea. These include treatments that use increased temperature, e.g., with the use of masks or apparatus treatments, such as manual facial cleansing with wapozon, diamond microdermabrasion, and dermapen. Any procedures that may intensify erythema or increase the temperature of tissues are contraindicated. Generally, strong massages should be avoided, but lymphatic drainage and gentle stroking movements are acceptable. 

Cosmetologists should propose skincare that has a soothing effect on erythema and aims to constrict blood vessels. The cosmetics used in the treatments should be non-irritating and have a low degree of fragrance. Apparatus treatments are to ensure the proper penetration of active ingredients into the skin, as well as provide a protective and filmogenic effect. The ingredients contained in cosmetics should also rebuild the lipid barrier. Recommended ingredients can include extracts of sweet almonds, wheat germ, oils (avocado and evening primrose), and jojoba. A soothing effect can be obtained using soy extract, d-panthenol, allantoin, or peptides. The anti-inflammatory effect can be ensured by using hops, cornflower, green tea, linseed, and chamomile. Cosmetics with probiotics, prebiotics, and synbiotics are new to the market, but have been proven to stimulate the growth of natural microflora, as well as smooth and nourish the skin [48,56,68,69].

The limitation of this research is the nonsystematic nature of this review. The selection of the articles included was based on the author’s professional experience gained in the treatment of rosacea. For this reason, bias cannot be excluded, and gaps in the current knowledge were not identified.

## 8. Conclusions

Treatment of rosacea poses challenges for the treating physician and patients; therefore, the role a cosmetologist plays in the treatment of rosacea is of high importance. The implementation of appropriate treatment and proper office and home care are the basis for obtaining good outcomes. A cosmetologist is more accessible to patients and can help to implement healthy daily habits, including skin care and eating habits, as well as support and mediate good communication between the patient and patient’s treating physician, thereby improving compliance and ensuring long-term satisfactory outcomes.

## Figures and Tables

**Table 1:** Selected studies on the composition of microflora in people with rosacea.

Study	Study Conclusions
Chen et al. [41]	Using next-generation sequencing, a decrease in the colon microbiome was observed in patients and a simultaneous increase in colonization by *Rabdochlamydia*, *Bifidobacterium*, *Sarcina*, and *Ruminococcus* and a decrease in colonization by *Lactobacillus*, *Megasphaera*, *Acidaminococcus*, *Haemophilus*, *Roseburia*, and *Clostridium*.
Nam et al. [42]	The study showed increased colonization by *Acidaminococcus*, *Megasphaera*, and *Lactobacillus*.
Agnoletti et al. [43]	The study showed a relationship between the maculopapular form of rosacea, SIBO syndrome, *Helicobacter pylori* infection, and the presence of the erythematous phase of acne.
Yun et al. [44]	The study indicated the possibility of altering the blood microbiome in the course of rosacea and other dermatological diseases. The presence of *Chromatiaceae*, *Fusobacteriaceae*, and *Rheinheimer* was demonstrated in patients with rosacea.
Thompson et al. [45]	The skin analysis in the course of rosacea showed an increased amount of *Actinobacteria*, including *Serratia marcescens* and *Cutibacterium acnes* compared to patients with acne vulgaris.
O’Reilly et al. [46]	In people with the presence of erythematous changes with telangiectasias and maculopapulars, an increased amount of *Demodex folliculorum* was found.
Dahl et al. [47]	High levels of *Staphylococcus epidermidis* were found in patients with rosacea. This microorganism is able to produce proteins at higher temperatures in patients with rosacea, which influences its pathogenic role.

## Data Availability

Not applicable.
